# Effects of workload and distance traveled on psychological resilience and values among emergency healthcare workers

**DOI:** 10.55730/1300-0144.5994

**Published:** 2024-11-28

**Authors:** Ramiz YAZICI, Efe Demir BALA, Ayşe Fethiye BASA KALAFAT, Eyüp SARI, Hüseyin MUTLU, Hakan GÜNER, Hilmi KAYA, Rabia Birsen TAPKAN, Utku Murat KALAFAT, Serkan DOĞAN

**Affiliations:** 1 Department of Emergency Medicine, İstanbul Kanuni Sultan Süleyman Training and Research Hospital, University of Health Sciences, İstanbul, Turkiye; 2 Department of Pediatrics, Gülhane Faculty of Medicine, University of Health Sciences, Ankara, Turkiye; 3 Department of Emergency Medicine, Faculty of Medicine, Aksaray University, Aksaray, Turkiye; 4 General Directorate of Emergency Health Services, Ministry of Health, Ankara, Turkiye

**Keywords:** Burnout, paramedics, emergency medical services, resilience, ambulances

## Abstract

**Background/aim:**

Emergency medical services (EMS) personnel face significant challenges in their daily work, which can lead to burnout and affect their mental well-being. This study aimed to investigate how workload and distance traveled, as specific aspects of EMS work, influence the psychological resilience and values of EMS personnel. The Connor–Davidson Resilience Scale (CD-RISC) and Spiritual and Humanistic Value Scale (SHVS) were utilized to assess psychological resilience and values, respectively.

**Materials and methods:**

A cross-sectional study was conducted among EMS personnel in Ankara, Türkiye, utilizing a questionnaire survey distributed between 23 and 30 May 2024. The study was approved by the Medical Research Scientific and Ethical Evaluation Board of Ankara Bilkent City Hospital. A total of 293 EMS personnel participated after providing informed consent and approval. The participants were categorized into 3 groups based on their case/distance ratio. We used the CD-RISC and SHVS to compare psychological resilience and values between the groups.

**Results:**

Significant differences were observed according to various sociodemographic and work-related variables among the participating EMS personnel. Younger personnel tended to handle more cases, while older personnel traveled longer distances on average (p < 0.001). Education level and experience also influenced workload allocation, with less experienced individuals and those with lower education levels handling more cases (p < 0.001). No significant differences were found in CD-RISC and SHVS scores between the considered groups; however, we identified significant differences based on sex (p = 0.005) for the CD-RISC and based on age (p < 0.05) and experience (p < 0.05) for the SHVS.

**Conclusion:**

This study highlights the complex relationship among workload, sociodemographic factors, and psychological resilience/values in EMS personnel. Older, more experienced personnel tended to exhibit higher resilience and values scores, suggesting greater adaptation and satisfaction. Understanding these dynamics can aid in developing targeted interventions to prevent burnout and promote the well-being of EMS personnel.

## 1. Introduction

Emergency medical services (EMS) personnel are often at the frontline of healthcare. Their long working hours and the nature of their jobs can burden their mental health. This burden may increase over time, leading to burnout and ultimately resulting in a loss of workforce [1]. EMS is a demanding career option, described as the bridge between the general public and healthcare, creating a stressful work environment where personnel deal with high levels of responsibility and insecurity issues [2].

Burnout was described by Freudenberger as a psychological syndrome that involves the three dimensions of emotional exhaustion, depersonalization, and reduced personal accomplishment [3]. After burnout was defined and awareness about burnout among medical staff increased, different instruments and tools were proposed to evaluate and measure the development of burnout, such as the commonly used Copenhagen Burnout Inventory and Maslach Burnout Inventory [4,5]. Certain sociodemographic factors can affect the development of burnout among EMS personnel, including age, marital status, increased workload, and night shifts [2–6]. Understanding the process of burnout requires understanding and evaluating the sociodemographic and other factors that can affect the mental health of EMS personnel. One way to understand the immense workload of EMS personnel and how it affects them as individuals is to present them with anonymous questionnaires addressing workload and work.

Alongside the factors that are work related and variable such as distance traveled and average daily case numbers for each participant in the present study, we also collected information on the participants’ marital status, living arrangements, education, and occupations so that we could evaluate the nonvariable factors that can contribute to burnout.

Another aspect of our study is psychological resilience. Various definitions have been given in different studies. The earliest studies on psychological resilience focused on people who were enthusiastic about life despite their negative living conditions and those who manage to overcome adversity by adapting to their situation [7]. Psychological resilience is defined as the ability of people to overcome and adapt to negative conditions even when they are challenged with a difficult crisis [8]. Those with inadequate coping mechanisms and resilience skills could be affected far more negatively than their colleagues during stressful events [9]. Thus, it is important to understand if the EMS personnel who face these kinds of events each day are equipped with adequate skills and resilience to manage such problems. In order to analyze these skills, we used the Connor–Davidson Resilience Scale (CD-RISC) to evaluate the psychological resilience of EMS workers.

We also included the evaluation of spiritual and humanistic values in our study. These values are defined important aspects of our lives, as they form the cornerstone of our interactions with the outside world. These values add meaning to our behavior by preparing a mental background for our actions. We used the Spiritual and Humanistic Value Scale (SHVS), developed by Güneş, to evaluate spiritual and humanistic values [10]. We included this scale in our study because we wanted to understand the mental approach of EMS personnel towards their work.

The present study aimed to understand how specific aspects of everyday EMS work affect average EMS personnel. We focused on workload in terms of daily case numbers and distance traveled in kilometers while on shift as two main elements of everyday EMS work. We chose these two parameters to focus on as they are the most reasonable scales to understand the intensity of an individual EMS personnel’s workload.

## 2. Materials and methods

### 2.1. Study design and setting

This was designed as a cross-sectional study based on a questionnaire survey conducted among EMS personnel in Ankara, Türkiye. The survey was conducted between 23 and 30 May 2024. The study protocol was reviewed and approved by the Medical Research Scientific and Ethical Evaluation Board of Ankara Bilkent City Hospital (Date: 22 May 2024, Decision No: TABED 1-24-285). All authors participating in the design of the study’s protocol followed the rules set out in the Declaration of Helsinki.

The study form and questionnaire were distributed to 372 EMS personnel working at 31 EMS stations in Ankara, along with an informed consent form, inviting them to participate in the study. The distribution and submission period for the forms was set between 23 and 30 May 2024. All participants who provided informed consent and completed the questionnaire within this time frame had their forms accepted for inclusion in the study. The exclusion criteria for the study were failure to submit the study form and informed consent within the specified time frame, not having worked at these stations during the whole of 2023, and a diagnosis of a psychiatric illness.

The participants were grouped based on the workload they were exposed to, according to the number of patients and the distance traveled. These groups were defined as those with the highest number of cases in a day, those who traveled the longest distance, and those with an average case/distance ratio. This categorization was performed after the survey data were collected, and the case/distance information was determined digitally based on EMS service points.

As stated above, we used the CD-RISC to assess the psychological resilience of the EMS workers. The CD-RISC was developed in 2003 by Connor and Davidson to analyze and assess psychological resilience, especially in people with posttraumatic stress disorder [11]. The original scale contains 25 items and responses are scored using a 5-point system that ranges from “not true at all” to “true nearly all of the time.” Besides the original 25-item version of the CD-RISC, there are also 2-item and 10-item versions called CD-RISC 2 and CD-RISC 10, respectively [12,13]. The 25 items of the CD-RISC each had a possible score of 4 and a total score of 100. Higher scores indicate higher psychological resistance to adversity. The literature on the CD-RISC continues to expand as the scale is translated into different languages and is used to assess the psychological resilience of different sociodemographic groups. In our study, we used the Turkish adaptation of the 25-item CD-RISC validated by Karaırmak [14]. The Cronbach’s alpha value for the Turkish CD-RISC was 0.89 in Karaırmak’s study [14], while in our study it was 0.90 (Table 1).

In addition to the CD-RISC, we used the SHVS, developed by Güneş, to assess the spiritual and humanistic values of the EMS personnel [10]. This scale was originally developed to assess tendencies towards spiritual and humanistic values in adolescents. It was tested among university students as well. The SHVS was designed based on the Turkish Ministry of National Education’s curriculum for values education. It is a 23-item questionnaire with 4 dimensions, namely spiritual values, love, awareness, and respectfulness. Answers are scored using a 5-point system that ranges from “very appropriate” (5 points) to “not appropriate at all” (1 point). Possible total scores range from 4 to 115, with higher scores indicating a higher tendency of alignment with the spiritual and humanistic values. The Cronbach’s alpha value for the SHVS was 0.84 in Güneş’s study [10], while in our study it was 0.92 (Table 1).

### 2.2. Statistical analysis

Data analysis was performed using IBM SPSS Statistics 27.0 (IBM Corp., Armonk, NY, USA). While evaluating the study data, in addition to calculating descriptive statistics (frequency, percentage, mean, standard deviation, median, minimum, and maximum), Pearson’s chi-squared test was used to compare qualitative data. The compliance of the data to the normal distribution was evaluated with the Kolmogorov–Smirnov test, skewness and kurtosis, and graphical methods (histogram, Q–Q plot, stem and leaf plot, and boxplot). The independent samples t-test and one-way analysis of variance (ANOVA) were used to compare normally distributed quantitative data between groups. Statistical significance was set at p < 0.05.

## 3. Results

In the comparisons between the considered groups, age, time spent at one’s current EMS station, education, experience, occupation, the occurrence of a transfer to a different station in 2023 and number of shifts in the new station, number of cases in 2023, and distance traveled in kilometers in 2023 all created statistically significant differences (p > 0.05). To identify the group or groups that caused those differences, multiple comparative tests were applied to the data groups (Table 2).

The participants were categorized into 3 groups according to whether they had the highest number of cases, traveled the furthest distances, or had an average distance/case ratio. Data analysis revealed that the three considered age groups differed between themselves. While the youngest participants tended to have the most cases, the oldest participants traveled the furthest distance on average (p < 0.001) (Table 2).

In terms of general prehospital tenure, the three groups had different values. The group with the most cases had the shortest duration and the group traveling the furthest distances had the longest duration (p < 0.001). A similar finding was observed for time spent at one’s current EMS station; the group with the most cases had spent the fewest years at their current stations and the group traveling the furthest distances had spent the most years at their current stations (p < 0.001) (Table 2).

The data analysis further showed that there was a significant difference between the group with the most cases and the group with the furthest distances traveled in terms of education. The group with the most cases had a lower level of education, while the group traveling the furthest distances had a higher education level (p < 0.001). In terms of total experience, it was seen that the group with the most cases had significantly less experience compared to the group traveling the furthest distances (p < 0.001) (Table 2).

Considering the occupations of the participants, there was no significant difference between ambulance and emergency care technicians among the three considered groups. This group of personnel was distributed evenly among the groups with the most cases, furthest distances traveled, and average distance/case ratios. Physicians, on the other hand, were more likely to be included in the group with average distance/case ratios compared to other occupations. Drivers were more likely to be included in the group with the most cases. Emergency medicine technicians and other occupations were more likely to be included in the group traveling the furthest distances (Table 2).

The participants in the group with the most cases were less likely to have been transferred to a different station in 2023 compared to those in the other groups (p < 0.001). For all other considered variables, no statistically significant difference was observed after the data analysis (Table 2).

In the comparisons of CD-RISC and SHVS scores, there was no statistically significant difference between the groups for general scores or the scores for specific dimensions (p > 0.05) (Table 3). However, upon comparing the scores according to sociodemographic variables, we found a statistically significant difference between men and women in terms of CD-RISC scores, as men had a higher mean score than women (p = 0.005). For other elements of the questionnaire, we did not find any statistically significant differences in CD-RISC scores between the groups (Table 3).

We also detected statistically significant differences in SHVS scores according to both age and total experience. To understand which groups caused the differences, we conducted multiple comparison tests. After the analysis, we found that participants aged >50 years had a higher mean SHVS score when compared to those aged <29 years or 30–39 years (p = 0.017). In terms of total experience, the group with >20 years of experience had a higher mean score compared to the other groups (p = 0.023). We did not find any other significant differences in SHVS scores between the groups (Table 3).

## 4. Discussion

In the present study, we aimed to analyze the sociodemographic data of EMS personnel based on their CD-RISC and SHVS scores to determine the average psychological status of these individuals and their humanistic and spiritual values.

We found that less experienced and younger personnel were more likely to have the most cases, while more experienced, educated, and older personnel were more likely to travel the furthest distances for their cases. This could be explained by the EMS command’s choice of teams for different cases, with younger and more energetic teams being assigned to more cases. This organizational preference, however, could cause a strain on the resilience of the EMS personnel, as mentioned by Gray et al. [15]. There is also a possibility of mobbing. As described by Picakciefe et al. in another study conducted in Türkiye, older and more experienced healthcare workers are more likely to display mobbing behaviors towards their younger and less experienced colleagues [16].

We found that participants with only undergraduate or high school education were assigned to more cases than their colleagues with graduate or postgraduate degrees. This may be attributed to the limited number of paramedics with graduate or postgraduate degrees, as noted in previous studies [17,18], with more educated staff being directed towards more complex cases rather than having more cases in total as an administrative policy. However, this approach could also cause more burnout and lower resilience, as having a highly demanding role is also shown as a possible factor for mental health problems in another study conducted by Harvey et al. [19].

Our data suggest positive correlations between SHVS scores and both age and experience. This could be explained by older professionals having had more time to adapt to their environments and develop the necessary coping skills to face everyday challenges. Ismail et al. reported similar results [20], although they also found a difference in CD-RISC scores in their data analysis.

As ours was a survey study involving a questionnaire, concerns could have arisen among the participants regarding their data and confidentiality. We tried to address such concerns by using an anonymous form to protect the participants’ confidentiality. There are also other limitations of our study to be noted. As the study aimed to evaluate the psychological well-being of the participants, their psychological state at the time of the questionnaire may have created bias in their answers.

Our study had some further limitations. As it was conducted only in Ankara, it may not reflect the resilience and burnout tendency of other EMS personnel across the country. We also had a limited time span to collect the data, which prevented us from follow up scoring for the participants after a certain time, to see if age or tenure truly affects the resilience and SHVS scores.

## 5. Conclusion

In the present study, we aimed to examine sociodemographic variables and their impacts on the psychological health of our participants for an assessment of the mental well-being and resilience of EMS personnel. We found that older and more experienced personnel tended to be more aware and adapted to their work and felt more satisfied about their professional and personal lives, with higher SHVS scores supporting this claim. We think that this result could be attributed to administrative preferences and tenure, as older personnel tend to have more experience in managing a challenging work environment after their first years, while younger personnel are trying to adapt. However, we also think that EMS organizations should be watchful of bullying and mobbing within their teams. Our findings could be used in the creation and evaluation of preventive measures against burnout among healthcare workers. Future research is needed to understand whether administrative preferences or experience is a better supporting factor for providing higher psychological resilience.

## Figures and Tables

**Table 1:** Cronbach’s alpha values of the scales.

		Cronbach’s alpha
CD-RISC		0.908
SHVS	Inner	0.876
	Outer	0.847
	General	0.920

**Table 2:** Comparisons between the groups.

Variables		Most cases (n=89)^1^	Furthest distances (n=98)^2^	Average distance/case ratio (n=106)^3^	p	Source of difference
Sex	Female	53 (59.6%)	54 (55.1%)	64 (60.4%)	0.720 a	--
	Male	36 (40.4%)	44 (44.9%)	42 (39.6%)		
Age		31.7±5.8	38.1±8.0	34.7±8.4	**<0.001** b	1 – 2 – 3
	≤29 years	34 (38.2%)	14 (14.3%)	24 (22.6%)	**<0.001** a	1 vs. 2
	30–39 years	46 (51.7%)	39 (39.8%)	58 (54.7%)		--
	40–49 years	9 (10.1%)	38 (38.8%)	17 (16.0%)		2 vs. 1 – 3
	≥50 years	0 (0.0%)	7 (7.1%)	7 (6.6%)		1 vs. 2 – 3
Prehospital tenure (years)		7.0±4.9	11.3±5.2	9.2±5.2	**<0.001** b	1 – 2 – 3
Time spent at current station (years)		6.9±5.0	10.8±5.4	9.0±5.3	**<0.001** b	1 – 2 – 3
Marital status	Married	61 (68.5%)	78 (79.6%)	70 (66.0%)	0.080 a	--
	Single	28 (31.5%)	20 (20.4%)	36 (34.0%)		
Education	High school	19 (21.3%)	8 (8.2%)	12 (11.3%)	**<0.001** a	1 vs. 2
	Undergraduate	48 (53.9%)	29 (29.6%)	41 (38.7%)		
	Graduate or postgraduate	22 (24.7%)	61 (62.2%)	53 (50.0%)		
Total experience (years)		6.4±4.7	12.1±7.0	10.0±7.0	**<0.001** b	1 – 2 – 3
	≤10 years	71 (79.8%)	37 (37.8%)	64 (60.4%)	**<0.001** a	1 – 2 – 3
	11–20 years	18 (20.2%)	50 (51.0%)	35 (33.0%)		2 vs. 1 – 3
	>20 years	0 (0.0%)	11 (11.2%)	7 (6.6%)		1 vs. 2 – 3
Living arrangements	Alone/roommate	11 (12.4%)	8 (8.2%)	9 (8.5%)	0.558 a	--
	Family/partner	78 (87.6%)	90 (91.8%)	97 (91.5%)		
Dependent family members	None	18 (20.2%)	14 (14.3%)	19 (17.9%)	0.553 a	--
	Partner	11 (12.4%)	9 (9.2%)	11 (10.4%)		
	Children	17 (19.1%)	33 (33.7%)	29 (27.4%)		
	Mother/father/sibling	12 (13.5%)	12 (12.2%)	18 (17.0%)		
	Partner and children	31 (34.8%)	30 (30.6%)	29 (27.4%)		
Occupation	Physician	3 (3.4%)	0 (0.0%)	7 (6.6%)	**<0.001** a	2 vs. 3
	Ambulance and emergency care technician	33 (37.1%)	27 (27.6%)	38 (35.8%)		--
	Emergency medicine technician	32 (36.0%)	54 (55.1%)	53 (50.0%)		1 vs. 2
	Driver	21 (23.6%)	9 (9.2%)	6 (5.7%)		1 vs. 2 – 3
	Other	0 (0.0%)	8 (8.2%)	2 (1.9%)		1 vs. 2
Were you transferred to another station in 2023?	No	2 (2.2%)	28 (28.6%)	22 (20.8%)	**<0.001** a	1 vs. 2 – 3
	Yes	87 (97.8%)	70 (71.4%)	84 (79.2%)		
	If yes, how many shifts did you work?	11.6±11.1	9.4±12.1	11.6±15.7	**0.031** b	--
Annual paid leave in 2023 (days)		19.6±12.3	19.1±17.4	18.0±12.9	0.726 b	--
Annual unpaid leave due to incapacity to work in 2023 (days)		10.2±14.3	7.6±15.1	6.1±16.2	0.180 b	--
Annual case numbers in 2023		1028.4±275.9	551.7±253.1	716.7±255.8	**<0.001** b	1 – 2 – 3
Annual distance traveled in 2023		18632.0±6691.2	31197.0±11451.8	18352.0±6644.3	**<0.001** b	2 vs. 1 – 2

a Pearson’s chi-squared test (n/%),

b One-way ANOVA (mean ± SD).

**Table 3:** Comparison of scores according to sociodemographic variables.

Variables		CD-RISC Total	SHVS		
			Inner	Outer	General
Group	Most cases (n=89)	25.2±8.7	3.5±0.8	3.0±0.9	3.3±0.8
	Furthest distances traveled (n=98)	25.0±7.7	3.5±0.6	3.0±0.7	3.3±0.6
	Average distance/cases ratio (n=106)	27.0±8.0	3.6±0.6	3.0±0.9	3.4±0.7
	**p** a	0.160	0.452	0.803	0.594
Sex	Female (n=171)	24.7±8.0	3.5±0.6	3.0±0.8	3.3±0.7
	Male (n=122)	27.4±8.0	3.6±0.7	3.1±0.8	3.4±0.7
	**p** b	**0.005**	0.587	0.260	0.393
Age	≤29 years (n=72)^1^	24.5±8.5	3.5±0.6	2.9±0.6	3.3±0.6
	30–39 years (n=143)^2^	25.7±8.3	3.4±0.7	2.9±0.8	3.2±0.7
	40–49 years (n=64)^3^	27.1±7.8	3.6±0.7	3.1±1.0	3.4±0.8
	≥50 years (n=14)^4^	27.6±5.0	4.0±0.4	3.6±0.8	3.8±0.5
	**p** a	0.234	**0.032**	**0.021**	**0.017**
	**Source of difference**	--	4 vs. 1 – 2	4 vs. 1 – 2	4 vs. 1 – 2
Marital status	Married (n=209)	25.7±8.3	3.5±0.7	3.0±0.8	3.3±0.7
	Single (n=84)	26.1±7.8	3.5±0.6	3.0±0.8	3.3±0.7
	**p** b	0.658	0.973	0.743	0.858
Education	High school (n=39)	26.1±8.3	3.4±0.8	3.1±1.0	3.3±0.8
	Undergraduate (n=118)	25.8±8.9	3.6±0.7	3.1±0.8	3.4±0.7
	Graduate or postgraduate (n=136)	25.7±7.4	3.5±0.6	2.9±0.7	3.3±0.6
	**p** a	0.973	0.590	0.156	0.547
Total experience	≤10 years (n=172)^1^	25.5±8.4	3.5±0.7	3.0±0.8	3.3±0.7
	11–20 years (n=103)^2^	26.0±8.2	3.5±0.7	3.0±0.9	3.3±0.7
	>20 years (n=18)^3^	27.6±5.3	3.9±0.3	3.5±0.7	3.8±0.4
	**p** a	0.566	**0.046**	**0.025**	**0.023**
	**Source of difference**	--	3 vs. 1 – 2	3 vs. 1 – 2	3 vs. 1 – 2
Living arrangements	Alone/roommate (n=28)	25.5±8.3	3.4±0.6	3.0±0.6	3.3±0.5
	Family/partner (n=265)	25.8±8.1	3.5±0.7	3.0±0.8	3.3±0.7
	**p** b	0.858	0.523	0.942	0.694
Dependent family members	None (n=51)	25.6±9.2	3.4±0.8	2.9±0.9	3.2±0.8
	Partner (n=31)	24.6±9.0	3.7±0.6	3.2±0.8	3.5±0.6
	Children (n=79)	26.1±7.2	3.5±0.6	3.1±0.8	3.3±0.7
	Mother/father/sibling (n=42)	24.9±8.0	3.5±0.6	3.0±0.7	3.3±0.6
	Partner and children (n=90)	26.4±8.2	3.5±0.7	2.9±0.8	3.3±0.7
	**p** a	0.762	0.384	0.390	0.417
Occupation	Physician (n=10)	27.5±2.9	3.5±0.5	3.1±0.5	3.3±0.5
	Ambulance and emergency care technician (n=98)	25.3±8.3	3.6±0.6	3.0±0.7	3.3±0.6
	Emergency medicine technician (n=139)	25.5±8.5	3.5±0.7	2.9±0.8	3.2±0.7
	Driver (n=36)	27.4±7.6	3.6±0.8	3.3±1.0	3.5±0.9
	Other (n=10)	28.1±5.0	3.8±0.3	3.4±0.7	3.6±0.4
	**p** a	0.511	0.471	0.079	0.225
Were you transferred to another station in 2023?	No (n=52)	25.6±7.1	3.5±0.6	3.0±0.7	3.3±0.6
	Yes (n=241)	25.8±8.3	3.5±0.7	3.0±0.8	3.3±0.7
	**p** b	0.844	0.855	0.866	0.980

a One-way ANOVA (mean ± SD),

b Independent samples t-test (mean ± SD).
